# Computer-assisted cognitive training in children with developmental disorders: a scoping review of available tools, clinical targets, and evidence gaps

**DOI:** 10.3389/fped.2026.1764054

**Published:** 2026-04-14

**Authors:** Maria Grazia Maggio, Paolina Ettorre, Alessandra Benenati, Irene Ciancarelli, Fulvia Di Iulio, Marco Iosa, Rocco Salvatore Calabrò, Giovanni Morone

**Affiliations:** 1 IRCCS Centro Neurolesi Bonino Pulejo, Messina, Italy; 2 Department of Life, Health and Environmental Sciences, University of L'Aquila, L'Aquila, Italy; 3 Department of Psychology “Renzo Canestrari”, University of Bologna, Bologna, Italy; 4 IRCCS Santa Lucia Foundation, Rome, Italy; 5 Department of Psychology, Sapienza University of Rome, Rome, Italy

**Keywords:** cognitive rehabilitation, computer-assisted cognitive training, developmental disorders, executive functions, neurorehabilitation, rehabilitation software, working memory

## Abstract

**Background:**

Computerized cognitive training (CCT) is increasingly used in pediatric rehabilitation; however, its application across developmental disorders remains heterogeneous in terms of targets, delivery models, and outcomes. This scoping review aimed to map the currently available CCT tools used in children with developmental disorders and to summarize their main characteristics, clinical targets, and evidence gaps.

**Methods:**

We conducted a scoping review in accordance with the PRISMA-ScR framework and registered the protocol on the Open Science Framework (OSF; DOI 10.17605/OSF.IO/9XQ5H). We searched peer-reviewed studies investigating CCT in children with developmental disorders and extracted data on device characteristics, target domains, training modalities, study design, and main findings.

**Results:**

Twenty-two studies describing 21 devices were included. Evidence was heterogeneous across diagnoses, intervention architectures, comparators, and outcome measures. The most consistent signal emerged in ADHD, where some programs reported improvements in working memory and selected executive-function outcomes. Evidence in learning-related and intellectual developmental conditions was more variable and device-specific, while the only ASD study identified did not show superiority over mock training.

**Discussion:**

CCT appears clinically attractive because of its adaptability, gamified delivery, and potential for home-based use; however, the current evidence base is uneven and does not support broad efficacy claims across developmental disorders. More disorder-specific studies with stronger comparators and ecologically valid outcomes are needed.

**Systematic Review Registration:**

https://osf.io/9xq5h, doi: 10.17605/OSF.IO/9XQ5H.

## 1 Introduction

Developmental disabilities (DD) can limit learning, language, physical abilities, or behavior. These limitations, caused by disorders of the developing nervous system, arise during infancy and childhood (1).

The United Nations Children's Fund (UNICEF) database noticed that about 240 million children worldwide suffer from DD, based on household surveys. The World Bank and the Global Burden of Diseases databases estimate this number to be around 290 million using statistical models (2).

According to the International Classification of Diseases (ICD-10), the major categories of neurodevelopmental disorders include Intellectual Disability, Specific Learning Disabilities, Hearing and Speech disorders, Specific DD of academic skills, Behavior disorders, Motor disorders, and Vision and Hearing disorders (3). In particular, intellectual disabilities are marked by intellectual difficulties and challenges in conceptual, social, and practical daily living abilities. Classification of severity ranges from “mild” to “profound” and indicates increasing support needs (4). According to DSM-5, specific learning disorders arise from persistent difficulties in reading, mathematics, and/or written expression, usually recognized during the school years (5). Among behavioral disorders, autism spectrum disorder (ASD) and attention-deficit/hyperactivity disorder (ADHD) are included. ADHD arises in childhood and is typically characterized by age-inappropriate inattention and/or hyperactivity/impulsivity (5). ASD is usually identified in early development and is characterized by differences in social communication together with restricted or repetitive patterns of behavior, interests, and/or sensory features, with variable impact on everyday functioning across individuals (5, 6).

Conventional treatments include pharmacological and non-pharmacological approaches. Pharmacological therapies are mainly intended to reduce symptoms and facilitate participation in behavioral and educational interventions. Non-pharmacological interventions are heterogeneous and diagnosis-specific, including behavioral, educational, language-based, academic, and family-centered supports (7–11). Many developmental disorders involve alterations in cognitive development and/or in the functional expression of cognitive skills in everyday life, including attention, working memory, language-related processing, executive functions, and learning (12–14). For this reason, cognition-oriented interventions may be clinically relevant in selected subgroups, although the presence, severity, and functional significance of cognitive difficulties vary across diagnoses and across individuals. Repeated structured cognitive practice may improve performance on trained tasks or closely related domains, but far-transfer and everyday functional gains remain variable across studies (15).

In the last decade, computerized cognitive training (CCT) has gradually emerged as a technology-enabled adjunct for the rehabilitation of developmental disorders (16, 17). CCT programs are designed to target relevant cognitive processes through repeated practice, adaptive task progression, and performance feedback, complementing broader non-pharmacological interventions (18). CCT for children with developmental disorders includes educational software, interactive exercises, serious games, and cognitive training applications. These tools can be delivered through engaging and personalized activities and may target cognitive, academic, behavioral, or socio-emotional skills. Digital intervention is becoming increasingly common in pediatrics, and the FDA authorization of EndeavorRx has further increased clinical interest in prescription digital therapeutics for ADHD (19, 20).

Across disorders, CCT typically operates through a common set of mechanisms: repeated practice of a predefined cognitive process, adaptive difficulty scaling, immediate feedback or reward, and relatively high training intensity delivered across multiple short sessions. The specific clinical target, however, differs by disorder. In learning disorders and language-related conditions, tasks often emphasize phonological discrimination, grapheme–phoneme mapping, decoding, and reading-related processes. In ADHD, programs more commonly train working memory, inhibitory control, sustained attention, and cognitive flexibility through adaptive game-like exercises. In intellectual developmental conditions, interventions tend to combine attention, memory, reasoning, and socio-emotional modules with simplified interfaces and closer supervision. In ASD, the evidence base identified here was limited to one randomized trial, so mechanistic inferences remain preliminary.

Recent literature suggests that the most consistent evidence for CCT currently comes from ADHD-focused programs, particularly for near-transfer outcomes such as working memory and selected executive functions (17). Additional reviews and recent applied studies in ADHD also support the relevance of gamified or computerized executive-function training (21, 22). Early exploratory reports had already described computer-based practice in learning settings (23), whereas more recent reviews of digital interventions for language and learning-related difficulties indicate a heterogeneous and still device-specific evidence base outside ADHD (24). These differences make it important to distinguish between feasibility, improvement on trained tasks, and broader functional transfer when interpreting the literature.

There are several different types of cognitive rehabilitation software, each with its own set of characteristics and exercises that allow the therapist to adapt the level of difficulty to the patient's abilities, potentially activating multiple cognitive domains simultaneously (25). These programs offer practical advantages for clinicians and families, such as providing a wide range of activities, enabling individualized programs, supplying feedback to the participant, adjusting task difficulty, and tracking progress over time (26, 27). Another advantage of CCT is that software-based interventions can be deployed flexibly in home-based or hybrid care pathways, which may improve accessibility and support continuity of care when supervision and digital infrastructure are adequate (28). Despite these strengths, these devices also come with limitations, including required digital skills, variable device reliability, and concerns related to excessive screen time (29). Technical issues such as software crashes or poor usability may also interfere with adherence, especially when supervision needs are high or routines are disrupted (30). Taken together, these features make CCT clinically attractive, but they do not by themselves establish efficacy across developmental disorders.

Accordingly, this scoping review aimed to map the currently available CCT tools used in children with developmental disorders, describe the cognitive processes they target and how they are delivered, and critically summarize the strengths, limitations, and evidence gaps of the available studies. We did not aim to estimate pooled efficacy across diagnoses, given the anticipated heterogeneity in populations, intervention architectures, and outcomes.

## 2 Evidence acquisition

To map the current landscape of CCT in developmental disorders, we conducted a scoping review in accordance with the PRISMA-ScR framework (31). The protocol was registered in the Open Science Framework (OSF; DOI 10.17605/OSF.IO/9XQ5H).

5wWe used the PICO (Population, Intervention, Comparison, and Outcome) model to operationalize the research question (32). The population consisted of children with developmental disorders. The intervention was any computer-assisted cognitive training tool targeting cognitive or related functional domains. Comparators included conventional interventions, sham conditions, or no intervention when present. The outcome of interest was not a pooled efficacy estimate but the mapping of devices, training targets, delivery modalities, and main reported findings.

## 3 Search strategy and selections

A review was conducted for all peer-reviewed articles using the following search terms/keywords: “computer-assisted training”, “cognitive rehabilitation”, “child”, and “cognitive developmental disorders”. A comprehensive search was performed in PubMed, Scopus, Cochrane, Embase, and Web of Science (see Table 1). All search results were imported into Rayyan and screened by two blinded reviewers (P.E. and A.B.) to minimize selection bias. After initial title and abstract screening, disagreements regarding inclusion or exclusion were resolved with two senior investigators (M.G.M. and G.M.). The selected articles were then reviewed in full text and summarized according to the predefined inclusion and exclusion criteria.

**Table 1:** Search strategy across databases.

Database	Search terms/keywords
PubMed	Computer-assisted training; cognitive rehabilitation; child; cognitive developmental disorders.
Scopus	Computer-assisted training; cognitive rehabilitation; child; cognitive developmental disorders.
Cochrane	Computer-assisted training; cognitive rehabilitation; child; cognitive developmental disorders.
Embase	Computer-assisted training; cognitive rehabilitation; child; cognitive developmental disorders.
Web of Science	Computer-assisted training; cognitive rehabilitation; child; cognitive developmental disorders.

The inclusion criteria were: (i) children with Cognitive, Hearing, Speech, and Behavioral Developmental Disorders; (ii) studies that described or investigated the use of Computer-Assisted Cognitive Rehabilitation; (iii) publication in the English language; and (iv) publication in a peer-reviewed journal.

We excluded articles describing theoretical models, methodological approaches, algorithms, and basic technical descriptions. We also excluded: (i) children diagnosed with motor developmental disorders, as classified under ICD-10 criteria for developmental motor disorders (ICD-10 code F82) (3); (ii) animal studies; and (iii) conference proceedings or reviews.

## 4 Data extraction

**Figure 1:**
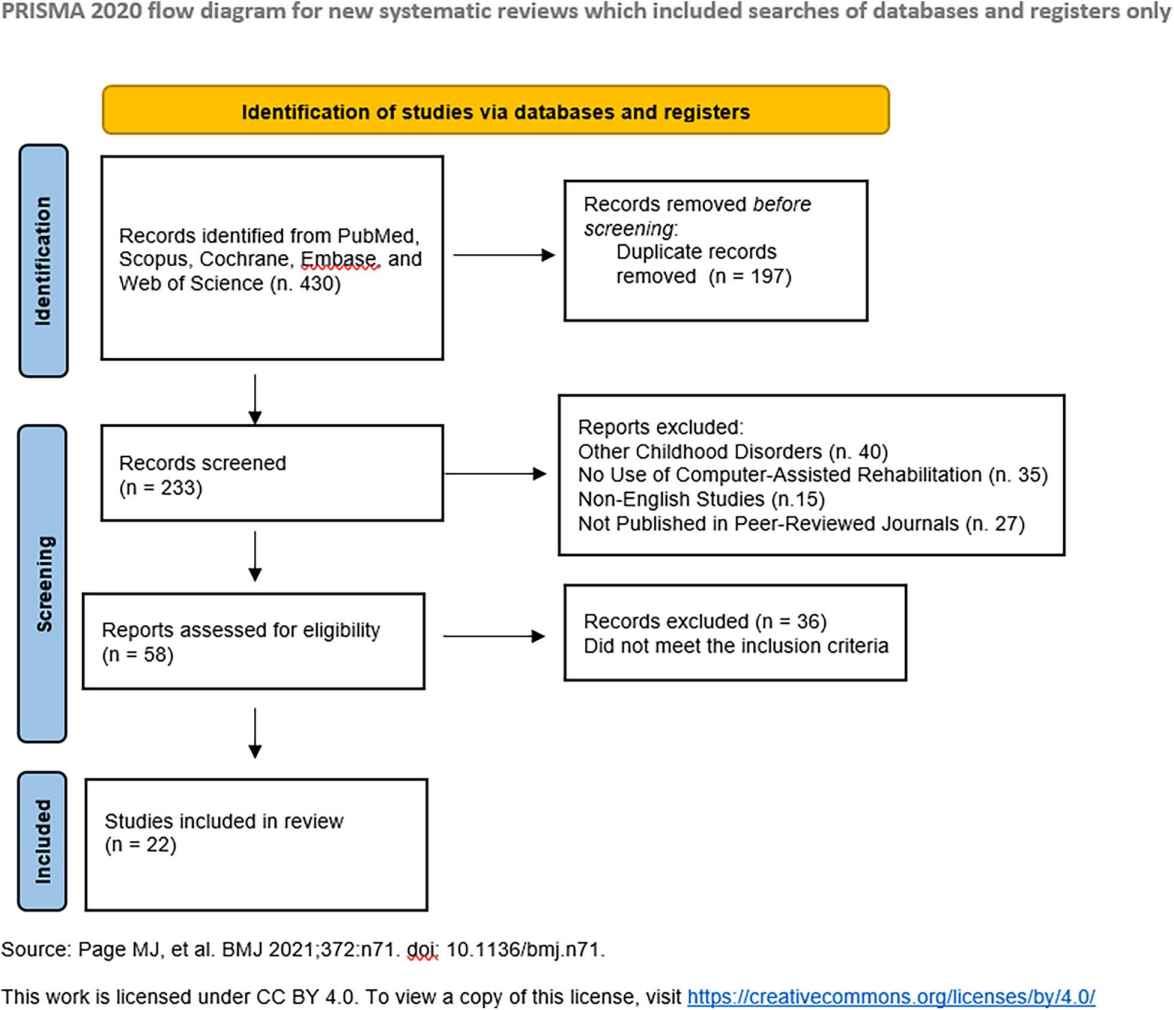
Flow chart selection process.

Following a preliminary search, 430 articles were found. After removing duplicates, 233 articles remained. Furthermore, 175 articles were excluded because they did not meet the inclusion criteria (related to other disorders in children such as acquired brain injury, or epilepsy). The remaining 58 articles were submitted to the externally supervised reviewer (M.G.M., R.S.C., and G.M.) because there was 98.3% agreement between the two reviewers and some of the included articles were of questionable inclusion for both (A.B., and P.E.). After in-depth analysis, only 22 articles were relevant to the research objectives and met the criteria. For more detail see Figure 1.

**Figure 2:**
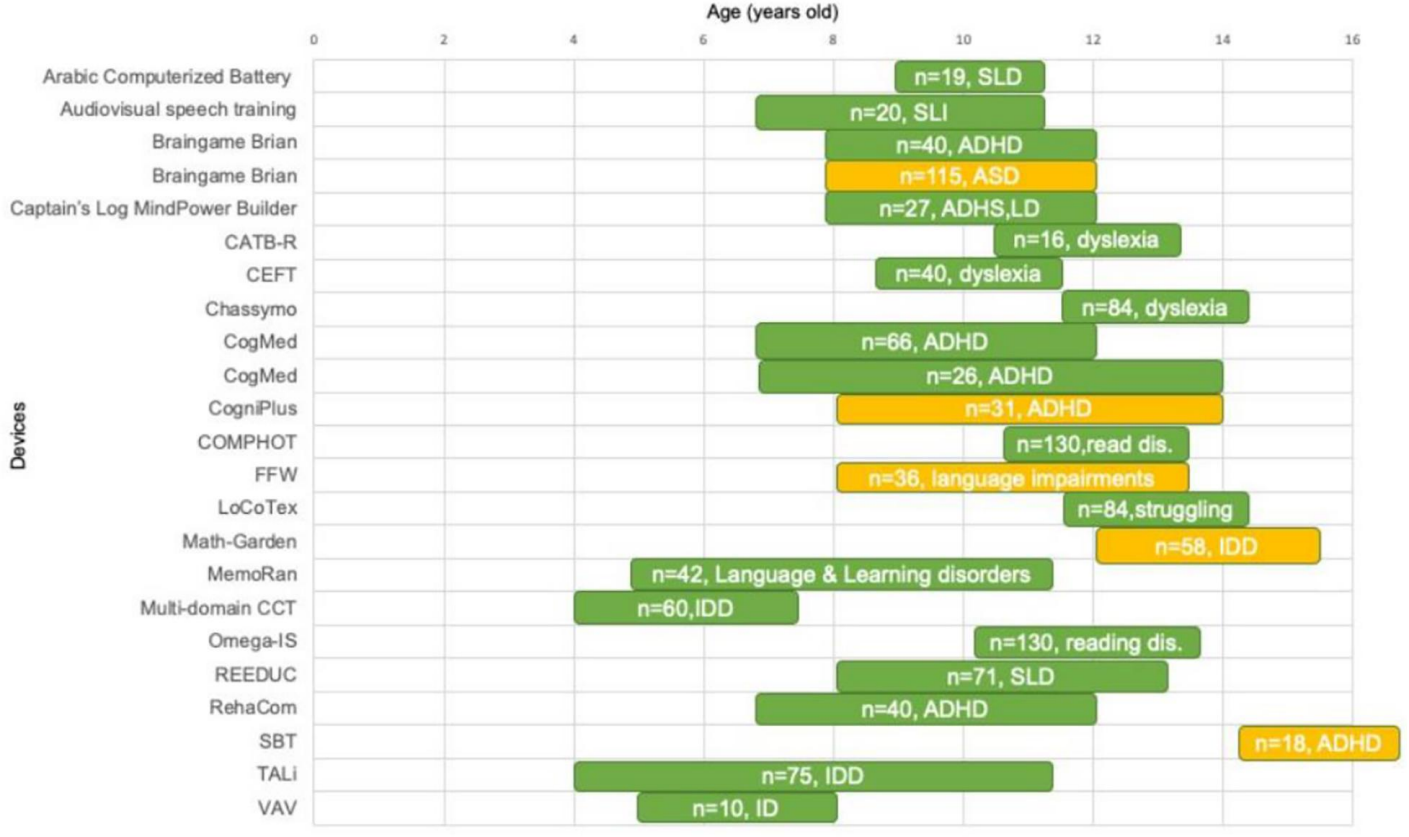
The figure shows the different devices and describes the age range in which they were used, by how many subjects, and by what disease. Positive results are highlighted in green; in yellow if they were not different from the control group. Legend: ADHD, Attention Deficit Hyperactivity Disorder; CATB-R, Computerized Cognitive Abilities training battery for Reading; CEFT, Computerized Executive Functions Training; FFW, FastForWordH; ID, Intellectual Disability; IDD, Intellectual and Developmental Disorders; read.dis., Reading Disabilities; SBT, Scientific Brain Training; SLD, Specific Learning Disorders; SLI, Specific Language Impairment; struggling readers, children with Reading difficulties; TALi, Training Attention and Learning Initiative; VAV, Vis-à-Vis.

## 5 Evidence synthesis

The literature search led to 21 cognitive devices used in the field of developmental disabilities. The results were organized into two tables. In Table 2, the devices are listed with their main characteristics, the studies in which they were used, and the reference clinical populations. Table 3 summarizes the study designs, training modalities, and main findings regarding treatment efficacy. All devices considered were software-based. The mapped landscape also included one ASD-focused platform evaluated in a randomized trial (33). Some devices had been developed exclusively for research purposes, whereas others were commercially available or required a professional license. Only one platform, Omega-IS, was described as free and open-source (Figure 2).

**Table 2:** List of devices and their main characteristics, main studies, and clinical populations on which they have been tested.

Device	Device description	License	Type of feedback	Adaptive training approach	Studies	Population	Cognitive domains
Computerized Executive Functions Training (CEFT) (Basharpoor, Seif, Daneshvar) Type: Software	Computerized Executive Functions Training (CEFT) is a CCT software specific for Iranian dyslexia children. It is made up of four computer games designed around three core executive function processes: working memory, inhibitory control, and cognitive flexibility. Tasks consist in choosing a different letter among similar letters, select images and words with the same meaning, arranging jumbled letters into a meaningful word within a specific time limit and click and identify 10 new words presented one by one.	Developed for research purposes. Not commercially available.	Real-time auditory feedback on errors; Recorded results at the end of the session	Not reported	Basharpoor et al., (2024)	Dyslexia	Working memory, inhibitory control, cognitive flexibility
Computerized Cognitive abilities training battery for reading (CATB-R). (Farghaly, El Tallawy, Ramadan, Abdelraso, Omar, Eltallawy, Mohamed) Type: Software	Computerized Cognitive abilities training battery for reading (CATB-R) is a computerized training battery used to develop and enhance cognitive skills useful to learning to read. It is made up of game-like tasks with multiple levels of increasing difficulty. The child has to master the current level in order to pass to the next one.	Developed for research purposes. Not commercially available.	Not reported	Available	Farghaly et al., (2022)	Dyslexia	Text reading comprehension, visual discrimination, auditory discrimination, phonological awareness, visuospatial skills, audio-visual correspondence, auditory memory
Arabic Computerized Battery (Farghaly, Ahmed, El-Tallawy, Elmestikawy, Badry, Farghaly, Omar, Hussein, Salamah, Mohammed) Type: Software	The Arabic Computerized battery aims to train cognitive skills for children with specific learning disabilities. It is based on training visual cognitive skills, such as visuo-spatial abilities, auditory cognitive skills, such as auditory discrimination, and a writing training program. The software combined phonological and orthographic presentations of the 28 alphabetic Arabic letters to help children understand the phoneme-grapheme relationship. Moreover, the computerized program had an audio-visual system which consisted of explicit and systematic instructions for transcription of the phonemic element of each one of the alphabetic Arabic letters to their corresponding grapheme.	Developed for research purposes. Not commercially available.	A follow-up sheet was constructed for each student to evaluate the time and errors for each item of the training program.	Available	Farghaly et al., (2018)	SLD	Visual cognitive skills, auditory cognitive skills
REEDUC (Exercices informatisés) Pépin M., Québec: Microlude, Inc., 1988 Type: Software	REEDUC is a computerized training program. It involves several different cognitive exercises, designed to enhance cognitive functions by progressive training. It runs on IBM-pc computers with 80-88 processors connected to a network. Performances are recorded at the end of every working session.	Developed for research purposes. Not commercially available.	Not reported	Available	Talbot et al. (1992)	SLD	Visual memory, perceptual speed, spatial visualization, recognition of forms, spatial orientation, mental arithmetic.
Audiovisual speech training (Heikkilä, Lonka, Meronen, Tuovinen, Eronen, Leppänen, Richardson, Ahonen, Tiippana) Type: Software	The programme included various tasks requiring phonological decisions. Spoken words, pictures, letters and written syllables were used as training material. Spoken words were presented either as audiovisual speech (together with the talking face), or as auditory speech (voice alone).	Developed for research purposes. Not commercially available.	Feedback points were yellow smileys.	Not reported	Jenni Heikkilä et al. (2018)	Specific language impairment (SLI)	Phonological skills, short-term memory, picture naming, attention and verbal motor skills
FastForWordH (FFW), Carnegie Learning, Inc., 1996 Pittsburgh, PA Type: Software	FFW involves a set of “games”, each training different aspects of language (e.g., auditory discrimination, phonological awareness, sentence comprehension).	Commercially available. Free trial available on subscription.	A correct response is followed by an additional icon and a cheerful sound; an incorrect response leads to a “sigh” noise and no additional icon.	Difficulty is adjusted until a threshold level is reached at which the child achieves 75% correct.	D. V. M. Bishop et al. (2006)	specific and non-specific language impairments	Language, grammatical comprehension, with and without the type of speech modification used in FFW.
MemoRan Anastasis Società Cooperativa Sociale, 2014, Bologna BO Type: Tele-rehabilitation software	MemoRan consists of 8 different exercises, to be carried out on a PC or tablet, which involve the exposure and timed naming of stimuli, such as various figures that are presented in matrices.	Full version available on payment. Free trial of 30 days available.	Adjusting the intervention to the subject's capacities	Available (self-adaptive exercises)	Agnese Capodieci et al. (2022)	Language and Learning Disorders	Exercises of inhibition, cognitive flexibility and updating in working memory
Chassymo (Ecalle & Magnan, 2010; AdepRio) Type: Software	Chassymo is grapho-syllabic training software program. This software program encourages grapho-syllabic word processing and is designed to promote word decoding skills.	Developed for research purposes. No longer available for purchase.	Corrective feedback: the word is written, and the syllable previously seen and heard is highlighted in green.	Not reported	Anna Potocki (2015)	Struggling readers	Word decoding skills
LoCoTex (Ecalle, Potocki, Jabouley & Magnan, 2014; AdepRio) Type: Software	LoCoTex is a text comprehension training software program. This software distinguishes between two aspects of text comprehension, namely literal comprehension and inferential skills.	Developed for research purposes. No longer available for purchase.	Corrective feedback followed by a brief explanation.	Not reported			Literal comprehension and inferential skills
Omega-IS Heimann, Lundälv, Tjus & Nelson, 2004-2006 Topic Dos Hb, Meloton Hb, Tomas Tjus Psykologbyrå & SuperImpact Images, Inc (USA) (https://omega-is.se/) Type: Software for Windows, Mac and Linux platforms.	Omega-IS is a computerized comprehension-oriented reading program that uses a top-down strategy focusing on both word-level and sentence-level language comprehension through interactive multimedia exercises.	Free and open-source software	Immediate feedback is provided at the end of each exercise	Available	Fälth et al. (2013)	Reading disabilities	Reading skills
COMPHOT Ferreira, Gustavvson, Rönnberg (2003) Linköping university: Swedish Institute for Disability Research Type: Software	COMputerized PHOnological Training (COMPHOT) is a phonological training programme which focuses on phonemes, phonemes linked to letters, word segments and words. The exercises are mainly sound-based: the child has to pair the picture with the corresponding word, or has to combine segments of words. It is made up of four exercise sections: rhyme, position, addition and segmentation.	Developed for research purposes. Not commercially available.	Immediate feedback after each task with the inclusion of personal high-score lists	Available			Reading comprehension, word decoding, sight word reading, phonological awareness
Training Attention and Learning Initiative (TALi) TALi Health Pty Ltd, Australia, 2011 (http://www.talihealth.com.au/) Type: Software app	TALi is a cognitive computerized training programme which enhances attention skills by completing different tasks through an interaction with a touch screen tablet. The first activity focuses on selective attention: children are required to locate a target fish among a series of distractor fishes. The second activity trains sustained attention: children have to monitor a gold coin on the screen and select it only when it stops moving. Activities three and four train aspects of attentional control: in the third activity, children have to make a response (left or right) based on the direction the elephant is facing, without considering other elephant distractors; in the fourth activity, children are required to tap the screen when an elephant appears, but not when a lion appears	Full version available on payment	Animated coins and stars after correcting completing each session	Available	Kirk et al. (2016)	IDD	Selective attention, sustained attention, cognitive flexibility (conflict resolution and response inhibition).
Multi-domain computerized cognitive training (Wu, Peng, Li, Deng, Huang, He, Tu, Cao, Huang) Type: Tablet software	This Multi-domain computerized cognitive training consists of four blocks (visual perception, attention, memory, and reasoning), each including two trials (eight trials in total). Each exercise trains a specific cognitive ability which is impaired in children with IDD. For instance, in the spatial perception trial the child has to classify different categories of objects, such as vegetables, and put them back where they should be stored (vegetables inside a basket).	Developed for research purposes. Not commercially available.	Immediate feedback according to the child's performance (as stickers and fireworks/thumbs up on the screen as the task reward)	Available (difficulty increases 80% correct answers)	Wu et al. (2023)	IDD	Visual perception, attention, working memory, and reasoning
Vis-à-Vis (VAV) Glaser, Lothe, Chabloz, Dukes, Pasca, Redoute, Eliez, 2012 Type: Software	VAV is composed of three “modules” or main cognitive domains: Focus on the Eyes, Emotion Recognition and Understanding, and Working Memory.	Developed for research purposes. Not commercially available.	Not reported	Exercises become progressively more difficult throughout the 12 weeks	Bronwyn Glaser et al. (2012)	Intellectual disability	Focus on the Eyes, Emotion Recognition and Understanding, and Working Memory.
Math Garden University of Amsterdam (Klinkenberg et al., 2011) Prowise Learn, Budel, NL, 2009 (http://www.rekentuin.nl;http://www.mathsgarden.com/). Type: Software	It is a web-based computer-adaptive application for practicing and monitoring math skills.	Commercially available. Free trial available.	After a correct answer participants were rewarded a virtual coin, but they lost this number of coins from their collection if the answer was incorrect.	Available	Brenda R.J. Jansen et al. (2012)	Adolescents with mild to borderline ID	Math skills and executive functions
CogniPlus, Schuhfried GmbH, Vienna, Austria, 2008. (http://www.schuhfried.com) Type: Software	CogniPlus is a training battery software for specific cognitive abilities, and its content is based on the Vienna Test System. This software provides an efficient combination between treatment and evaluation, exploiting the role of therapeutic assessment. CogniPlus can also adapt to patients’ abilities by offering exercises based on performance.	Commercially available. Professionals use only. No free trial available.	Electronic point reward system, with a visual display of progression over time.	Available	Minder et al. (2019)	ADHD	Attention, inhibition, working memory
RehaCom®, HASOMED GmbH, Magdeburg, Germany, 1997. www.rehacom.com Type: Software with specific tool for human-computer interaction.	RehaCom is a cognitive rehabilitation program consisting of 20 modules with several subsections that are selected and used by the therapist according to the needs of the participant. The RehaCom hardware has a special keyboard with large buttons, which limits the interference of motor and coordination impairment and expertise in computer use. Online monitoring is also available for the therapist to assess the function of the participant.	Commercially available. Professionals use only. No free trial available.	Progress can be saved, but no direct feedback is provided at the end of exercises.	Available	Mozaffari et al. (2022)	ADHD	Attention, response control, selective and divided attention, response control to visual and acoustic information.
CogMED QM training, Pearson Company, Stockholm, Sweden, 2011. (http://www.cogmed.com) Type: Software	The CogMed is a computer-based solutions software for cognitive training of attention problems caused by poor working memory (WM). Use of Cogmed requires a computer and/or tablet with speakers, stable broadband internet connection of 0.5 Mbit/s or higher, Adobe Flash plugin version 10.0 or later, and minimal hard drive space to store results. It is programmed for Mac, PC, and Android devices. It is most commonly run online through the Cogmed website where users are provided a unique ID and password.	Commercially available. Professionals use only. Available on subscription.	Coach online	Available	Aitana Bigorra et al. (2016) (CWMT)	ADHD	WM
					Chloe T. Green et al. (2012)	ADHD	Verbal and visuospatial WM span tasks.
Scientific Brain Training (SBT) program HAPPY neuron Pro, 2012, Campbell, CA Type: Website platform	Scientific Brain Training is a commercially available program for adults. It is made up of several exercises for professionals who want to provide effective cognitive stimulation.	Commercially available. Free trial available	A congratulation screen appears after completing a workout.	Difficulty is adjusted automatically to the user's performance.	Aida Bikic (2017)	ADHD	Visual and spatial skills, working memory, attention, visual short-term memory,vigilance, concentration.
Braingame Brian, Gaming & Training Foundation, Netherlands, 2011 (http://en.gamingandtraining.nl//) Type: Software	Braingame Brian is a computerized game training of visuospatial working memory, cognitive flexibility and inhibition. The software consists of 25 training sessions of about 40-50 min each. Each session contains two blocks of the three training tasks. At the end of each block, the game's difficulty is automatically adjusted to the child's performance. Each completed block of training results in extra powers for Biran, the main character of the game, or improvements of the game world, in order to enhance children's motivation.	Commercially available. Professionals use only.	Not reported	The difficulty level of the training task is automatically adjusted to the child's level of performance.	S. Van Der Oord et al. (2014)	ADHD	Visuospatial WM, inhibition, cognitive flexibility
					Marieke de Vries et al. (2015)	ASD	WM-training, a flexibility-training, or a mock-training
Captain's Log MindPower Builder; BrainTrain Inc., North Chesterfield (VA, USA) (https://www.braintrain.com/) Type: Software	This software is designed to help improve memory, attention, perception, reasoning, planning, judgment, general learning, and overall executive functioning.	Commercially available. Professional use only.	Built-in reward system	Available	Antonio Carlos Farias et al. (2017)	ADHD-LD	Attention, reaction/inhibition, stimulus reaction/time, and stimulus reaction/fields, memory, visuo-spatial memory, logic skills.

SLD, Specific Learning Disorders; SLI, Specific Language Impairments; LD, Learning Disabilities; ASD, Autism Spectrum Disorders; ADHD, Attention Deficit Hyperactivity Disorder; IDD, Intellectual and Developmental Disorders; CG, Control group.

**Table 3:** List of devices, the types of studies that were conducted, how the device was used and the main findings in terms of treatment efficacy.

Device	Studies	Sample characteristic	Type of study	Training protocols	Treatment duration location supervision	Outcome measures & efficacy	Main findings	Adverse events and missing schedule therapy
Computerized Executive Functions Training (CEFT) (Basharpoor, Seif, Daneshvar)	Basharpoor et al., (2024)	40 children with dyslexia (20 CG) aged from 9 to 11	RCT	The experimental group underwent CEFT sessions over 4 weeks, while the control group played a neutral computer game during 12 sessions.	12 sessions over 4 weeks (CEFT for experimental group, neutral computer game for control group)	Word reading (*p* = 0.035); word chains reading (*p* = 0.020); picture naming (*p* = 0.045); text comprehension (*p* = 0.030); word comprehension (*p* = 0.020); non-words and pseudo-word (*p* = 0.034); letter fluency (*p* = 0.014)	CEFT improves the ability of children with dyslexia in word reading, word chains reading and non-word and pseudo-word reading.	10 participants were excluded from the study because they failed to follow the instructions (*n* = 3) and did not attend all sessions (*n* = 7). So, the final sample size reached 40 (20 per group).
Computerized Cognitive abilities training battery for reading (CATB-R). (Farghaly, El Tallawy, Ramadan, Abdelraso, Omar, Eltallawy, Mohamed)	Farghaly et al., (2022)	16 2nd grade primary school students with dyslexia	Single group study	Training sessions with CATB-R	Five sessions per week for two months. Each session lasted for 90–120 min. Rehabilitation was carried out in the neuroepidemiology centre at Asyut University under the supervision of a therapist	Reading Comprehension (*p* = 0.0001); auditory discrimination (*p* = 0.002); phonological awareness (*p* = 0.0001); visuo-spatial skills (*p* = 0.002); audio-visual correspondence (*p* = 0.004); total reading disability score (*p* = 0.0001)	There was a significant improvement on the scores of different cognitive skills. This improvement in reading cognitive abilities was associated with a significant improvement in reading achievement as evaluated by ART	Not reported
Arabic Computerized Battery (Farghaly, Ahmed, El-Tallawy, Elmestikawy, Badry, Farghaly, Omar, Hussein, Salamah, Mohammed)	Farghaly et al., (2018)	19 students with SLD aged from 9 to 11	Single group study	Training sessions tailored according to each child's previously diagnosed cognitive skill deficits.	Three sessions per week, for a total of 54 sessions in 18 weeks (2 weeks for writing training skills, 8 weeks for visual skills, and 8 weeks for auditory skills). Every session lasted from 90 to 120 min. The rehabilitation program was carried out in the Neuroepidemiology Research Center of the Faculty of Medicine, Assiut University, with the supervision of three neurologists and two staff members from the Faculty of Education.	Total visual closure (*p* = 0.011); total spatial relations (*p* = 0.038); total visual memory (*p* = 0.004); total whole-part relationships (*p* = 0.034); total phonological awareness (*p* = 0.001); total auditory discrimination (*p* = 0.001); total auditory memory (*p* = 0.003); total auditory comprehension (*p* = 0.001)	Participants improved in phonological awareness. This, together with other improved auditory and visual cognitive abilities, was reflected in improved academic achievement (reading, writing and mathematics).	Not reported
REEDUC (Exercices informatisés) Pépin M., Québec: Microlude, Inc., 1988	Talbot et al. (1992)	71 students with SLD (11 CG) aged from 8 to 13	Experimental study	The experimental group was subjected to six exercises to train visual memory, perceptual speed, spatial visualization, recognition of forms, spatial orientation, and mental arithmetic. Mental arithmetic and spatial visualization exercises were practiced for twice as long. The control group received no intervention.	8-hour training divided into one session per week, each session lasting about 30 min. Training sessions took place in groups of 12 students, at school, and were conducted by two psychologists and a teacher.	Mental arithmetic (*p* = .02)	Training has been shown to have a significant effect on mental calculation skills. Therefore, practicing computerized mental calculation exercises can facilitate the automation of basic arithmetic operations, such as addition, subtraction, and multiplication.	Not reported
Audiovisual speech training (Heikkilä, Lonka, Meronen, Tuovinen, Eronen, Leppänen, Richardson, Ahonen, Tiippana)	Jenni Heikkilä et al. (2018)	20 children aged from 7.2 to 10.8 years with SLI: 10 children in the audiovisual training group (AV), 10 children in the auditory training group (A)	Experimental study	The audiovisual group (A) underwent the training with the programme utilizing audiovisual speech. The auditory group (A) underwent the same training but with auditory speech only, so that when a spoken word was presented, a black computer screen was presented instead of the talking face.	Six weeks, 5 days a week, 10–15 min per day, on schooldays, supervised by two speech therapists.	Repetition of Nonsense Words test (AV group) (pw = .006)	Children who underwent the audiovisual speech training programme improved in the Repetition of Nonsense Words, whereas children in the auditory training group did not. So, audiovisual speech might be more effective than auditory speech in training phonological skills in children.	Not reported
FastForWordH (FFW) Carnegie Learning, Inc., 1996 Pittsburgh, PA	D. V. M. Bishop et al. (2006)	Thirty-six children with SLI participated in the study, aged from 8 to 13 years.	RCT	Children 8 to 13 years were randomly assigned to three groups: Group S (*n* = 12) responded to reversible sentences in a computerized game, using speech stimuli with pauses before critical phrases. Group M (*n* = 12) had the same stimuli acoustically modified to lengthen and amplify dynamic portions of the signal. Group U (*n* = 9) was an untrained control group	20 training sessions, each lasting 15 min. In general, training sessions took place on consecutive schooldays, supervised by School staff.	Testing session (*p* < .001)	This study offered no support for an auditory perceptual account of children's grammatical impairments.	Three children (one from group M and two from group S)
MemoRan Anastasis Società Cooperativa Sociale, 2014, Bologna BO	Agnese Capodieci et al. (2022)	24 children with SLD, 18 children with language disorders (LD), aged from 5 to 11 years.	Multicentre study	42 children: 24 with SLD and 18 with LD	15–25 min daily, atleast 3–4 times a week, for a period of three months. The training was conducted at home supervised by parents	Word dictation (DDE-2–errors) (*p* < 0.005); Alce reading speed (*p* < 0.05); Alce–reading accuracy(*p* < 0.05); NEPSY-II–verbal fluency correct responses(*p* < 0.05); RAN–response time (*p* < 0.05); PRCR-2–SD4 errors(*p* = 0.06); NEPSYII–inhibition rapidity (*p* = 0.09); NEPSY-II –inhibition accuracy (*p* < 0.001); Digit span backward (*p* = 0.05); BAF-listening span test–n.words (*p* < 0.05)	Improvements in reading and writing tests	No child dropped out of the training
Chassymo (Ecalle & Magnan, 2010; AdepRio)	Anna Potocki (2015)	84 students (mean age 13 years) divided in 4 group: poor decoders-poor comprehenders (PDPC), poor decoders-normal comprehenders (PDNC), normal decoders-poor comprehenders (NDPC) and normal decoders-normal comprehenders. Each group of readers was further subdivided into two subgroups: One received decoding training using Chassymo, the other comprehension training using LoCoTex.	Experimental study	The participants were subdivided into four contrasted groups based on their scores in the silent word reading and listening comprehension tasks	30 min per day, 4 days a week, over a period of 4 weeks.	Word identification, (*p* < .05); Reading fluency (*p* < .01, only significant for the children trained with the Chassymo); listening comprehension (*p* < .001).	The students trained with the grapho-syllabic software improved primarily in the reading fluency task	Not reported
LoCoTex (Ecalle, Potocki, Jabouley & Magnan, 2014; AdepRio)							The students trained with the comprehension software showed improved performance in listening and reading comprehension.	
Omega-IS Heimann, Lundälv, Tjus & Nelson, 2004–2006 Topic Dos Hb, Meloton Hb, Tomas Tjus Psykologbyrå & SuperImpact Images, Inc (USA) (https://omega-is.se/)	Fälth et al. (2013)	130 students in Grade 2. 100 students with reading difficulties were divided into 4 groups: phonological training (*n* = 25), comprehension training (*n* = 25), combined training (*n* = 25), ordinary special instructions (*n* = 25). 30 students with no reading difficulties received no treatment (CG)	Longitudinal RCT	Children in the phonological training group received COMPHOT training; children in the comprehension training group received Omega-IS training; children in the combined training received both COMPHOT and Omega-IS training; children in the ordinary special instructions group underwent lessons with a special education teacher.	25 one-to-one training sessions, each lasting 15–25 min, with a special education teacher, at school	Comprehension training: WM (*p* < .05). Phonological training: RAN (*p* < .05); verbal fluency (*p* < .05). Combined training: nonverbal intelligence (*p* < .05); RAN (*p* < .05); WM (*p* < .01).	All groups improved their reading skills. However, combined training showed stronger improvements in decoding, reading comprehension, and non-word reading. These gains persisted over a 1-year follow-up period.	Not reported
COMPHOT Ferreira, Gustavvson, Rönnberg (2003) Linköping university: Swedish Institute for Disability Research								
Training Attention and Learning Initiative (TALi) TALi Health Pty Ltd, Australia, 2011 (http://www.talihealth.com.au/)	Kirk et al. (2016)	75 children aged 4 to 11 years old with IDD (37 CG)	RCT	The experimental group underwent 25 sessions of TALi on a tablet, in which task difficulty was automatically adjusted on a level by level basis. The control group underwent a nonadaptive control training programme	25 sessions, each of the duration of 20 min, once a day, 5 times a week for a 5-week period, at home, under the supervision of a parent/guardian.	Attention training (p_b_ < .05) post-training and follow-up	Participants in the experimental condition showed improvement in selective attention performance compared to children in the control condition. These improvements were maintained 3 months after training.	15 (4 CG) children did not meet compliance criteria (completing 15 out of 25 training sessions). Moreover, two participants were lost to follow-up testing (CG).
Multi-domain computerized cognitive training (Wu, Peng, Li, Deng, Huang, He, Tu, Cao, Huang)	Wu et al. (2023)	60 Children aged 4 to 6.5 years old with IDD (30 CG)	Single-blind RCT	Children in the experimental group underwent training sessions with the tablet, whereas children in the control group received a one-to-one training. All training sessions were arranged according to the individual characteristics of each child	Five weeks training, five times a week, for 20 min each time, under a therapist's supervision.	FSIQ (pw < 0.01), arithmetic and vocabulary of VIQ (pw < 0.01), ADQ (pw < 0.01): subdomains communication (*p* < 0.01) and socialization (*p* < 0.015)	The multi-domain CCT can significantly enhance the intellectual performance and adaptive functioning of children with IDD. The improved intellectual function is positively connected with improved adaptive functioning	10 children (5 CG) dropped out during intervention and in the 3 - months follow up
Vis-à-Vis (VAV) Glaser, Lothe, Chabloz, Dukes, Pasca, Redoute, Eliez, 2012	Glaser et al. (2012)	Ten children with idiopathic developmental delay (three girls, seven boys) aged between 7 and 10 years old (M age 5–8 years 6–9 months)	Single group study	The exercises are individually randomized within the first two (one and two) and the last two (three and four) of the four weekly practice sessions to ensure that each session is completed in a different order for each participant and so that the first and second sessions of each exercise fall at the beginning and end, respectively, of the participant's remediation week.	Four 20-min computerized sessions per week for 12 weeks supervised by an adult at school.	Module Focus on the Eyes: Session Number (*p* = .001), Emotion (*p* = .001), interaction of Emotion X Session Number (*p* = .001); Module Emotion Recognition and Understanding: Session Number (*p* = .001), Emotion (*p* = .001), Session Number 3 Emotion interaction (*p* = .001); Working Memory module: Sheep: (*p* = .001), Simon: (*p* = .001), between total weighted correct scores and session number: (*p* = .001).	Subjects improved on all three modules during training and on emotion recognition and nonverbal reasoning post-VAV.	One child was excluded from our statistical analyses because he left school before the third evaluation
Math Garden University of Amsterdam (Klinkenberg et al., 2011) Prowise Learn, Budel, NL, 2009 (http://www.rekentuin.nl;http://www.mathsgarden.com/)	Jansen et al. (2012)	Fifty-eight students (31 boys) aged between 12 and 15 years old, were divided into control and experimental groups (*N* = 29 in each group)	RCT	Exercises about addition, subtraction, multiplication, and division games.	4 times a week for 5 weeks, during school time and at home. Each session lasted approximately 10 min.	Training on math skills: main effect on time: subtraction *p* < .001, addition *p* = 0.13, multiplication *p* = .001, division *p* = .045. Training on FE: verbal WM *p* = .040, inhibition *p* = .003	Participants in both groups improved on all math domains except addition, but improvement did not differ between the experimental and the control group	Not reported
CogniPlus, Schuhfried GmbH, Vienna, Austria, 2008. (http://www.schuhfried.com)	Minder et al. (2019)	31 children with ADHD aged from 8 to 14 years old	Single group study	Training was tailored according to each child's skill deficits, by choosing from four out of ten CogniPlus tasks.	30 sessions, 45–60 min each, over 10 to 12 weeks. Training was conducted in a separate room at the participant's school (*n* = 13) or at an outpatient clinic (*n* = 18).	Divided attention (*p* < .05), flexibility (*p* < .05), Corsi (*p* < .05), Conners-3 (*p* < .01), BRIEF (*p* < .01)	A significant reduction in parent-rated ADHD symptoms and executive deficits assessed by the BRIE*F* test was observed. However, the response to neuropsychological treatment was less than expected. The training tasks used may not be adequate to produce improvements in the cognitive performance of children with ADHD.	Not reported
RehaCom®, HASOMED GmbH, Magdeburg, Germany, 1997. (http://www.rehacom.com)	Mozaffari et al. (2022)	40 children from 7 to 12 years old with ADHD divided in two groups: 20 children in the experimental group and 20 in the CG	RCT	The experimental group was treated by RehaCom program; the CG group had only the routine treatment (Ritalin)	Ten 45-minutes sessions during 5 weeks (2 sessions per week)	Auditory Attention (*p* > 0.05); Visual Attention (*p* > 0.05) post 5 weeks.	Therapeutic effect on both auditory and visual response control, but not on attention.	Not reported
CogMED QM training, Pearson Company, Stockholm, 2011 Sweden, (http://www.cogmed.com).	Bigorra et al. (2016) (CWMT RoboMemo® 2005, Cogmed Cognitive Medical Systems AB, Stock-holm, Sweden),	66 children between 7 and 12 years old with ADHD; 36 experimental group, 30 CG	RCT	The experimental group underwent CWMT RoboMemo® which consisted of visuospatial, auditory, and location memory and tracking of moving visual objects as WM tasks; The CG (non-adaptive training) engaged in the MegaMemo, which consists of the same WM tasks as the experimental group but without the adjustment for difficulty.	Each training session included 90 trials and had a duration of 30–45 min. Participants attended 5 sessions per week over a 5-week period for a total of 25 sessions.Training was conducted at home, under the supervision of a family member.	WM subscale (*p* = 0.01); plan/organize subscale (t = −2.02, df = 4, *p* = 0.05); meta-cognition index (t = −2.25, df = 4, *p* = 0.03); ADHD symptoms composite score (t = −2.69, df = 4, *p* = 0.01); Significant improvements in functional impairment (t = −2.43, df = 4, *p* = 0.02)	The strongest effects were observed on primary outcome measures EFs scales assessed by both parents and teachers, especially long-term effects; Long-term far-transfer improvements on ADHD symptoms, especially in metacognition, WM and planning skills	Not reported
	Green et al. (2012)	26 children (18 males; age, 7 to 14 years old); 12 in the active training condition and 14 in the placebo control condition	Randomized, double-blind, placebo-controlled design	The Cogmed training includes 10 verbal and visuospatial WM span tasks. Some tasks are both auditory and visual in nature, requiring cross-modal processing; In the placebo control-nonadaptive condition, the same tasks were used, but the difficulty level remained low throughout all of the training sessions.	Each participant was required to complete 90 trials of WM Cogmed tasks a day for 25 days. 40 min per session. Researchers instructed parents to supervise their children as they each performed the tasks.	WISC WMI composite (*p* = 0.02)	Significant effect of WM.	1 participant withdrawing from the placebo and 3 from the adaptive training condition.
Scientific Brain Training (SBT) program HAPPY neuron Pro, 2012, Campbell, CA	Aida Bikic (2017)	18 children with ADHD (9 in the cognitive training group and 8 in the active placebo group), mean age of 15.6 years, 76.5% boys.	Randomized double-blinded trial.	The intervention group used a selection of beta-exercises from the Scientific Brain Training (SBT) program; The control group played a common version of the game Tetris.	They were asked to play at home for half an hour a day, 5 days a week for 7 weeks, supervised by their parents.	APQ: Interest (*p* = 0.668), Value (*p* = 0.543), Choice (*p* = 0.286); SBT group: visual sustained attention (*p* = 0.0051). placebo Tetris group: of spatial working memory (*p* = 0.0417).	SBT showed a significant pre–post intra-group beneficial effect on two outcomes of sustained attention; Tetris had a significant positive pre–post intra-group effect on spatial working memory. No significant differences were found between groups on any measure.	1 child refused to participate. No adverse events were reported.
Braingame Brian, Gaming & Training Foundation, Netherlands, 2011 (http://en.gamingandtraining.nl//)	Van Der Oord et al. (2014)	40 students aged from 8 to 12 years old with ADHD (22 CG).	RCT	The experimental group underwent 25 training sessions consisting in three tasks of WM, inhibition and cognitive flexibility. The control group, after a 6 weeks waiting, underwent the treatment as well.	25 training sessions of about 40 min each, over a period of 5 weeks. The training was carried out at home, with parents’ supervision.	Cognitive flexibility (*p* = 0.01); inhibition (*p* = 0.01); WM (*p* = 0.01); DBDRS-P IA (*p* = 0.01); DBDRS-P H/I (*p* = 0.01)	Children in the training condition showed significant reductions in parent-rated ADHD behaviors and improvement in executive functions compared with children in the wait-list condition. Moreover, these improvements were maintained after a 9 weeks’ follow up.	3 children in the experimental condition did not meet the criterion of having completed at least 20 of the 25 training sessions
	De Vries et al. (2015)	115 children aged from 8 to 12 years old with ASD, divided into three training groups: 40 WM training, 37 flexibility training, 38 mock-training.	RCT	In each intervention condition, all EF tasks were performed. In the WM-training condition, WM tasks were set to be with increasing difficulty, while the other tasks remained at a low level. In the mock-training, all the tasks remained at a low level.	25 training sessions during six training-weeks. The training was carried out at home, with parents’ supervision. During this period, parents were contacted weekly about the child's progress.	Time effect on Corsi-BTT (*p* < .001); gender-emotion switch-task (*p* < .01).	Participants in all conditions improved in WM, cognitive flexibility, attention, and on parent's ratings, but not in inhibition.	Six children dropped out after pre-training. Then, twenty-five children did not complete 25 sessions (9 WM, 10 flexibilities, and 6 mock-training).
Captain's Log MindPower Builder; BrainTrain Inc., North Chesterfield (VA, USA) (https://www.braintrain.com/)	Farias et al. (2017)	Two groups of children aged from 8 to 12 years old with ADHD were configured: group A, participants medicated with immediate-release methylphenidate 20 mg/day (*n* = 19); and group B, unmedicated participants (*n* = 8).	Experimental study.	Both groups received these exercises: 1) attention-skills module – scanning reaction/inhibition, stimulus reaction/time, and stimulus reaction/fields; 2) conceptual/memory skills module – conceptual, logical, size discrimination, and symbol display match; 3) visual motor skills module – visual categorization, visuospatial memory, and visual time response; and 4) logic skills module – conceptual, matching, and sequential logic.	One-hour sessions were conducted twice weekly over a period of 3 months (total of 24 sessions). Rehabilitation was carried out at the research institute.	Unmedicated: Memory power (*p* = 0.028); Problem solution (*p* = 0.018); Concentration power (*p* = 0.018); Visual processing (*p* = 0.018); Self-discipline (*p* = 0.018); Auditory processing (*p* = 0.018). Medicated: Memory power (*p* = 0.011); Problem solution (*p* = 0.003); Concentration power (0.004); Visual processing (*p* = 0.001); Self-discipline (*p* = 0.019); Auditory processing (*p* = 0.004).	Both groups showed significant improvements in memory, problem solving, concentration, visual and auditory processing, and self-discipline exercise performance with training.	Of 30 families who fitted the inclusion criteria and were contacted to take part in the study, 3 partecipants dropped out.

APQ, Activity Perception Questionnaire; ART, Arabic reading test; FSIQ, Full-scale Intelligence Quotient; VIQ, Verbal Intelligence Quotient; ADQ, Adaptive Developmental Quotient; RAN, Rapid Automatized Naming; WM, Working Memory.

### 5.1 Specific learning disorders

Numerous computerized cognitive devices have been developed for the treatment of specific learning disorders. Several were created exclusively for research purposes, whereas others were commercially available or accessible through tele-rehabilitation. The included studies varied markedly in target skills, training dose, and outcome measures, making cross-device comparisons difficult.

Among the research-oriented devices summarized in Table 2, Basharpoor et al. (34) showed that Computerized Executive Functions Training improved reading-related outcomes in children with dyslexia. The Computerized Cognitive Abilities Training Battery for Reading (CATB-R) also demonstrated significant improvement in reading-related cognitive abilities, which was associated with better reading achievement (35). Farghaly et al. (36) used the Arabic computerized battery and showed that children with specific learning disorders experienced increased phonological awareness, which positively correlated with improvements in reading, writing, and mathematics skills. Another tool developed exclusively for research, Audiovisual Speech Training, showed that integrating audiovisual stimuli was more effective than auditory-only speech training for improving phonological skills in children (37). A commercially available language-oriented platform, FastForWord, has also been evaluated in this area, although trained groups did not differ from untrained children in language or auditory outcomes (38). Similarly, Potocki et al. found that the Chassymo and LoCoTex programs effectively enhanced reading fluency, listening, and comprehension in children with language impairment (39). Talbot et al. demonstrated that REEDUC training facilitated the automatization of basic arithmetic operations such as addition, subtraction, and multiplication in children with learning disabilities (23). Another research-specific tool, COMPHOT, focused on phonological training. Fälth et al. found it effective in improving reading skills in children with reading disabilities (40). However, the same authors suggested that combining COMPHOT with Omega-IS yielded stronger improvements in decoding, reading comprehension, and non-word reading (40).

Among other commercially available devices is MemoRan, a software that allows therapy sessions to be conducted from home. Capodieci et al. showed that after three months of training, results in reading and writing tests improved significantly (25).

### 5.2 Intellectual developmental disorders

Four studies described CCT tools for children with intellectual developmental disorders. Participants ranged in age from 4 to 15 years. Training lasted 5 weeks in the multi-domain trial (15) and the TALi study (41), whereas Vis-à-Vis lasted 12 weeks (42) and Math Garden followed a 5-week schedule with shorter 10-minute sessions (43). The multi-domain program (15) and VAV (42) required therapist supervision, whereas TALi (41) and Math Garden (43) could also be conducted at home.

The two devices developed exclusively for research purposes are the Multi-domain computerized cognitive training and the VAV. The first, used in a study by Wu et al. (15), was found to be effective in improving intellectual performance and adaptive functioning in children with IDD. The second, proposed in the study by Glaser et al. (42), demonstrated improvements in socio-emotional functioning and working memory.

The two commercially available devices are the Training Attention and Learning Initiative (TALi) and Math Garden. The TALi (41) led to improvements in selective attention performance, with these improvements persisting three months after the training. In contrast, Jansen et al. did not observe significant differences between the experimental group using MathGarden and the control group, which received no training (43). Both groups improved in all math domains except addition.

### 5.3 Attention deficit hyperactivity disorder

The cognitive devices used for treating children with ADHD are commercially available (44–50), although the effectiveness of CogniPlus (44), RehaCom (45), and Scientific Brain Training (48) has not been fully demonstrated. Participants in these studies ranged in age from 7 to 15.6 years.

In the study by Minder et al., CogniPlus was used to improve behavioral and neuropsychological outcomes of children with ADHD (44). Although a significant reduction in parent-rated ADHD symptoms and BRIEF executive impairments was noted, the improvements in neuropsychological outcomes, such as working memory and attention, were not as strong as expected. Conversely, Mozaffari et al. found that RehaCom specifically enhanced auditory and visual response control, but had no measurable effect on attention (45).

The CogMED QM training device was employed in studies by Bigorra et al. (46) and Green et al. (47). In the first study, training with this cognitive tool resulted in long-term, far-transfer improvements in ADHD symptoms, particularly in metacognition, working memory (WM), and planning skills (46). Significant improvements were also observed in executive function scales, as assessed by both parents and teachers. Green et al. similarly confirmed significant improvements in WM (47).

The effectiveness of other cognitive tools has also been explored. In the study by Bikic et al., Scientific Brain Training (SBT) was tested (48). Although no significant group differences were observed in cognitive or ADHD symptom measures following the web-based intervention, SBT showed notable pre-post improvements within the group in visual sustained attention and spatial WM.

Additionally, Braingame Brian was the cognitive tool used in the study by Van Der Oord et al., where children showed significant reductions in parent-rated ADHD behaviors and improvements in executive functions compared to those in the wait-list group (49). These gains were sustained at the 9-week follow-up.

In contrast, Farias et al. introduced Captain's Log MindPower Builder in their study, which focused on children with both ADHD and learning disabilities (50). Following training, all participants demonstrated improvements in attention, memory, certain executive functions, and academic performance, especially in mathematics, as well as a reduction in maladaptive behavioral traits.

### 5.4 Autism spectrum disorders

From the data collected in this scoping review, the only device identified for training children with ASD was Braingame Brian (33). In the study by de Vries et al. (33), the tool was used with children aged 8 to 12 years, and the training was conducted at home under parental supervision. The program lasted six weeks, with a total of 25 sessions. Participants across all conditions showed improvements in working memory, cognitive flexibility, attention, and parent-rated behaviors. However, the intervention groups did not significantly differ from the mock-training condition. Accordingly, the available ASD evidence was insufficient to support disorder-level conclusions about efficacy.

## 6 Discussion

The present scoping review underscores that CCT is gradually emerging as an innovative therapeutic option for children with developmental disorders. Rather than supporting a single efficacy estimate across diagnoses, the included literature maps a heterogeneous field of devices, targets, comparators, and outcomes. The clearest signal in the present review concerns ADHD-focused programs, particularly those targeting working memory and selected executive functions (44–50). These findings are broadly consistent with earlier Cogmed and preschool training literature, although transfer estimates remain sensitive to the choice of executive-function outcome measures (51–53).

A more critical reading of the included studies suggests that apparent benefits are influenced not only by the intervention target but also by study design. Many studies were small, single-center, or pilot investigations; several lacked active comparators, used wait-list or treatment-as-usual controls, or relied heavily on parent-rated outcomes, making expectancy effects and non-specific engagement effects difficult to exclude. In contrast, studies using more stringent comparators often reported more modest between-group effects, as illustrated by the ASD randomized trial (33) and by ADHD studies with limited between-group separation (44) or primarily within-group gains (48). This pattern suggests that CCT may be best viewed as a structured, engaging, and scalable adjunct for selected cognitive targets, rather than as a uniformly effective stand-alone treatment across developmental disorders.

When comparing these findings with research on other neurodevelopmental and cognitive disorders, the variability in results becomes more apparent. For instance, studies using CCT in children with learning disabilities (LD) have shown mixed outcomes. While Farias et al. (50) demonstrated that Captain's Log MindPower Builder improved attention, memory, executive functions, and academic performance in children with ADHD and LD, other research using similar devices in LD populations has not always found clear benefits, particularly in more complex cognitive domains such as problem-solving and abstract reasoning (48). These inconsistencies across different disorders highlight the need for more disorder-specific interventions and the potential limitations of generalizing results from one population to another.

Furthermore, CCT's effectiveness can vary depending on the cognitive domain being targeted. For example, Mozaffari et al. found that RehaCom improved auditory and visual response control but did not significantly affect attention, which suggests that not all cognitive processes are equally responsive to these interventions (45). This contrasts with evidence from adult traumatic brain injury populations, where a recent systematic review of randomized controlled trials suggested improvements in attention and executive-function-related domains after computerized cognitive rehabilitation (54). This difference may be due to the nature of the cognitive deficits associated with each disorder, with ADHD and ASD presenting more heterogeneous profiles that complicate intervention outcomes.

Although CCT is often discussed alongside other emerging technologies, comparative effectiveness between CCT, virtual reality, and non-invasive brain stimulation was not directly assessed in this review. Evidence from immersive VR or NIBS in other pediatric or neurological populations should therefore not be interpreted as evidence of superiority over CCT (55, 56). The most tangible practical advantage identified across the included CCT studies was scalability: several interventions were home-based, adaptive, and relatively easy to supervise by caregivers or clinicians, particularly in Cogmed studies (46, 47) and in the Captain's Log MindPower Builder study (50). In practice, the choice among digital rehabilitation strategies should depend on the target domain, child characteristics, supervision needs, acceptability, and available infrastructure rather than on assumptions of superiority.

Overall, the most consistent signal of benefit in this scoping review emerged in ADHD, especially for working memory and selected executive-function outcomes (44–50). Evidence in specific learning disorders and intellectual developmental conditions was more heterogeneous and study-specific. Evidence in ASD was insufficient for disorder-level conclusions, because only one randomized controlled trial was identified and it did not outperform mock training (33). Therefore, CCT should presently be discussed as a promising but uneven evidence area, with feasibility and mechanistic appeal exceeding the certainty of broad clinical effectiveness claims.

This review has important limitations. First, as a scoping review, it was designed to map the field rather than quantify pooled efficacy. Second, terminology across neurodevelopmental diagnoses and computerized interventions is highly variable; therefore, some disorder-specific studies may not have been retrieved under the selected cognition-oriented search terms. Third, the included literature was highly heterogeneous in sample size, intervention dose, comparator rigor, and outcome selection, limiting cross-study comparability.

## 7 Clinical applications and future perspectives

Based on the evidence mapped here, CCT appears most clinically promising in ADHD and in selected cognition-oriented interventions for learning-related disorders (34–40, 44–50). Its main clinical value lies in its adaptability, repeatability, and potential for home-based delivery under therapist or caregiver oversight, particularly in Cogmed studies (46, 47), in the Captain's Log MindPower Builder study (50), and within broader pediatric telerehabilitation models (57). This flexibility is particularly beneficial for children in remote areas or for families with limited access to specialized services. Moreover, digital performance tracking may help therapists monitor progress and adjust interventions over time. However, evidence for ASD remains too limited for specific clinical recommendations, because the only ASD randomized trial identified did not show superiority over mock training (33).

Regarding future perspectives, future studies should prioritize disorder-specific trial designs, active comparators, ecologically valid endpoints, and transparent reporting of adherence and transfer to everyday functioning. It will also be important to determine whether gamification mainly improves engagement or also produces durable clinical gains, as motivation-enhancing design features do not necessarily guarantee broader transfer (58, 59). Combination approaches with neurofeedback, brain-computer interfaces, or non-invasive brain stimulation remain promising but exploratory and were not directly assessed in the present review; recent evidence on non-invasive brain stimulation in ADHD suggests possible domain-specific benefits, although the overall evidence base remains heterogeneous and not yet definitive (60).

With these methodological improvements, CCT may become a more integrated component of individualized rehabilitation pathways, rather than a replacement for established multidisciplinary care.

## 8 Conclusion

This scoping review mapped the potential benefits and limitations of CCT in children with developmental disorders. The clearest, though still heterogeneous, signal of benefit was found in ADHD, particularly for working memory outcomes in Cogmed-related studies and selected executive-function results in Braingame Brian. Evidence in specific learning disorders and intellectual developmental conditions was variable across devices and study designs, while evidence in ASD remained insufficient for positive conclusions because the only identified randomized controlled trial did not show superiority over mock training. Accordingly, CCT should currently be interpreted as a promising and scalable adjunctive option whose effectiveness is likely to depend on the cognitive target, intervention architecture, comparator, and outcome selection.

Despite these limitations, the available studies suggest that CCT may serve as a useful adjunct within individualized rehabilitation pathways, particularly when the trained cognitive domain, delivery format, and outcome selection are carefully matched to the child's needs. Future research should prioritize stronger disorder-specific designs, active comparators, ecologically valid endpoints, and clearer reporting of transfer to everyday functioning.

## Data Availability

The original contributions presented in the study are included in the article/Supplementary Material, further inquiries can be directed to the corresponding author/s.
