# Supplementary figures and images for: Computer-assisted cognitive training in children with developmental disorders: a scoping review of available tools, clinical targets, and evidence gaps
Source: Front Pediatr. 2026 Apr 14;14:1764054. doi: 10.3389/fped.2026.1764054 (PMC13121333; doi:10.3389/fped.2026.1764054)

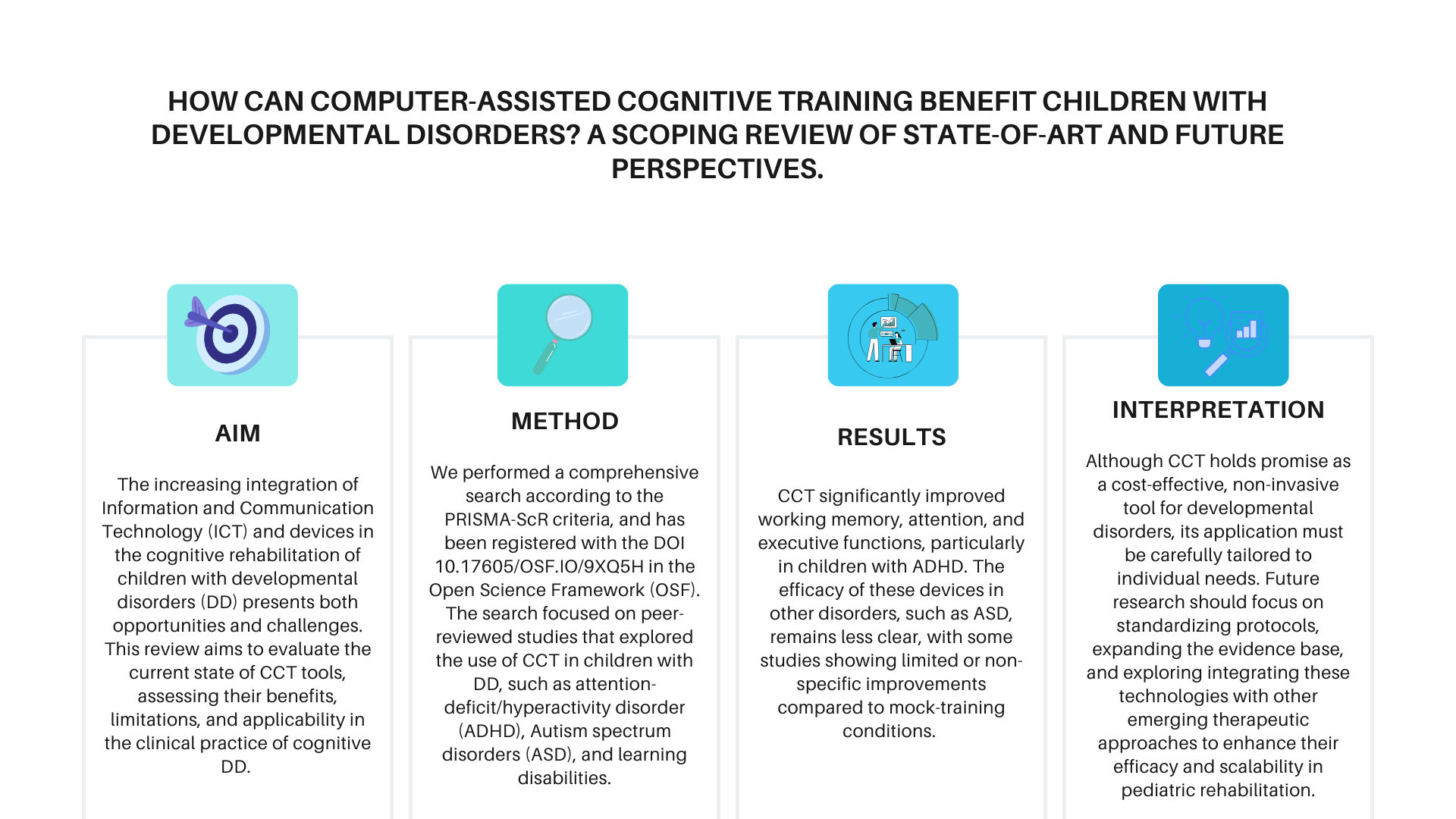

Supplement: Supplementary file 1 [file Image1.png]
